# Cement and Concrete Nanoscience and Nanotechnology

**DOI:** 10.3390/ma3020918

**Published:** 2010-02-03

**Authors:** Laila Raki, James Beaudoin, Rouhollah Alizadeh, Jon Makar, Taijiro Sato

**Affiliations:** National Research Council Canada, Institute for Research in Construction / 1200 Montreal Road, Bldg M-20, Ottawa, Ontario K1A 0R6, Canada; E-Mails: jim.beaudoin@nrc-cnrc.gc.ca (J.B.); rouhollah.alizadeh@nrc-cnrc.gc.ca (R.A.); jon.makar@nrc-cnrc.gc.ca (J.M.); taijiro.sato@nrc-cnrc.gc.ca (T.S.)

**Keywords:** nanotechnology, concrete, cement, C-S-H, controlled release, carbon nanotubes, nanoparticles

## Abstract

Concrete science is a multidisciplinary area of research where nanotechnology potentially offers the opportunity to enhance the understanding of concrete behavior, to engineer its properties and to lower production and ecological cost of construction materials. Recent work at the National Research Council Canada in the area of concrete materials research has shown the potential of improving concrete properties by modifying the structure of cement hydrates, addition of nanoparticles and nanotubes and controlling the delivery of admixtures. This article will focus on a review of these innovative achievements.

## 1. Introduction

Typical concretes consist of ordinary Portland cement (OPC), fillers such as sand, coarse aggregates, admixtures and water. This combination of materials allows concrete to be produced in a fluid form that can be pumped and moulded. The complex chemistry and physical structure of cement hydrates in concrete however mean that issues of fundamental science still need to be resolved. Research at the nanoscale has the potential to contribute to these debates and questions. Analysis at the nanoscale may provide further insight into the nature of hydrated cement phases and their interaction with admixtures, nanofillers and nanofibers. These interactions offer the possibility of modifying cement reactions, creating new surface chemistries (referred to as nanoscience), developing new products for the concrete industry (referred to as nanotechnology), and allowing a more controlled and ecologically friendly manufacturing route to cement and concrete.

## 2. C-S-H and C-S-H Composites

### 2.1. Calcium Silicate Hydrate (C-S-H)

#### 2.1.1. Formation and properties

The main product of the hydration of Portland cement is a nearly amorphous material − Calcium Silicate Hydrate (C-S-H) − that forms up to about 60% by volume of the paste. In cement chemistry, CaO, SiO_2_, and H_2_O are represented by C, S, and H respectively. The hyphens in C-S-H indicate indefinite stoichiometry and the hydrate is sometimes referred to as “C-S-H gel”. C-S-H is produced along with calcium hydroxide in the chemical reaction of the silicate phases (*i.e.*, β-C_2_S and C_3_S) with water. C-S-H is the principal binding agent in the cement paste and is responsible for its important properties such as strength and shrinkage. Resolving the structure of this material at the nano scale is an essential part of understanding and predicting its behavior. It is also important in the context of modification and development of novel C-S-H systems discussed in the next section.

The molar ratio of CaO to SiO_2_ (C/S ratio) in C-S-H is one of the main parameters in defining and controlling the properties of a calcium silicate hydrate system. This value varies from 1.2 to 2.1 in hydrated silicate phases and has an average of about 1.75 [1]. The C-S-H systems may be divided into low and high lime content categories partitioned by the C/S ratio of about 1.1 where chemical and physical properties change noticeably [2,3]. The state of water in a C-S-H system is also vaguely defined. Water can be present within the interlayer structure of C-S-H (either in the form of H_2_O or OH^-^). Water molecules can also be physically adsorbed on the surface of solid phases. Finally, the capillary pores (10–50 nm in diameter in well hydrated pastes and as large as 3–5 micrometeres at early ages) between C-S-H clusters can contain free water. Distinction of water states is not simple as the energy by which the water molecules are held in C-S-H varies over a wide range and may overlap for different locations [4].

There are several more ordered calcium silicate hydrates that are structurally related to the C-S-H. Tobermorite and jennite (with approximate stoichiometry of C_5_S_6_H_5_ and C_9_S_6_H_11_ respectively), for example, have a defined crystal structure and have been studied for many years as possible analogues to C-S-H. The reaction between lime and silica in excess water results in the formation of tobermorite-like and jennite-like systems most commonly known as C-S-H(I) and C-S-H(II). These hydrates can also be prepared through mixing sodium silicate and calcium salt in aqueous solution, although they are less crystalline. These phase pure materials are relatively easy to produce and are convenient for systematic research work on C-S-H.

#### 2.1.2. Nanostructural models of C-S-H

Study of the structure of C-S-H in Portland cement systems using X-ray diffraction is limited due to its poorly crystalline nature. Early research investigations were conducted using mainly surface area and density measurements, and, weight and length change isotherms in order to characterize this material [4,5]. In the last few decades, many new aspects of the C-S-H have been revealed with the advancements in the analytical techniques and application of new methods such as nuclear magnetic resonance (NMR) spectroscopy.

**Figure 1:**
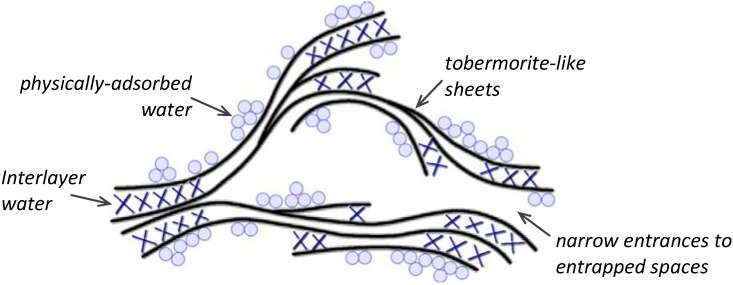
Simplified physical model for hydrated Portland cement.

The nanostructure of C-S-H has been the subject of much research, yet is still not clearly understood with suggested models ranging from colloidal to “layer-like”. One of the first physical models was proposed by Powers and Brownyard [6]. It describes C-S-H as a colloidal material. In this model the gel particles are held together mainly by van der Waals’ forces and the space between them is called “gel porosity” which is accessible only by water molecules. A more comprehensive model was developed later by Feldman and Sereda based on extensive experimental studies of hydrated cement systems [7,8]. The role of water in this model is explained in more detail and the changes in the mechanical properties of C-S-H related to water content can be easily described. The main feature of their model (shown in Figure 1) is concerned with the layered nature of the C-S-H. Structural roles that are assigned to the interlayer water of the C-S-H, exhibit irreversible behavior during the adsorption and desorption processes.

**Figure 2:**
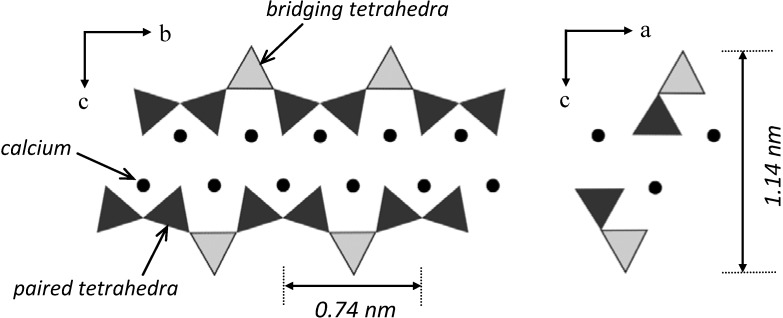
The schematic molecular structure of a single sheet of tobermorite. Circles: calcium atoms located at the center of Ca-O octahedra; Triangles show silicate tetrahedra ; OH^-^ attachments are not shown. Various tobermorite systems exist that vary in the interlayer distance, *i.e.*, 9, 11 and 14 Å tobermorites.

Advancement in experimental techniques, has led to the development of new models. Jennings’ colloid model features globules of about 5 nm in diameter for C-S-H and proposes the existence of intraglobular pores (IGP) and small gel pores [9]. The viability of using a layered model for the C-S-H in cement paste seems, however, also plausible according to recent work [10], which used helium inflow technique as a nanostructural probe along with XRD to follow the changes at the nano-level in the properties of C-S-H(I), a layered semi-crystalline material. The helium inflow results are analogous to those for C-S-H in hydrated Portland cement. They can be best explained by a layered model for C-S-H in cement paste. The Jennings colloidal model is essentially a hybrid where the ‘globules’are comprised of C-S-H layers. The layered model is incompatible with the colloid model in its explanation of physico-chemical and engineering behavior. The colloid model neglects the structural role of interlayer water in cement paste as evidenced by the corresponding behavior of synthetic C-S-H (I) and the more amorphous C-S-H present in the paste. A primary difference is rooted in the inability of the colloid model to separate the ‘reversible’ and ‘irreversible’ thermodynamic aspects of sorption phenomena. It is unable, for example, to rigorously explain elastic and viscoelastic behavior and their dependence on relative humidity. A schematic of the calcium silicate structure of a tobermorite layer is shown in Figure 2. It is suggested that the omission of bridging tetrahedra and further defects in the silicate chain in addition to the presence of calcium ions in the interlayer region can accommodate a variety of compositional changes for C-S-H systems [3,11,12].

#### 2.1.3. Mechanical properties

The mechanical properties of phase pure C-S-H systems have rarely been studied. The intrinsic modulus of elasticity of C-S-H appears to be independent of its C/S ratio and degree of polymerization [13]. Nanoindentation methods have been employed in order to study the elastic character of C-S-H nanoparticles [14]. Two types of C-S-H are suggested − low and high density. The low and high density C-S-H phases, with a volume fraction of 30 and 70% in hydrated OPC, have a mean stiffness of about 21.7 and 29.4 GPa, respectively. Work by the authors using dynamic mechanical analysis methods has shown that the complex stages of changes in the storage modulus (E’) and internal friction (tan δ) of phase pure C-S-H(I) on the removal of adsorbed and interlayer water is consistent with that for the C-S-H in OPC [15]. It has also been shown that the decrease in C/S ratio of C-S-H increases its stiffness.

Dynamic molecular modeling and free energy minimization techniques have also been used to estimate the elastic properties of C-S-H. It has been reported that the average Young’s modulus (E) increases with the increase in the C/S ratio of the C-S-H [16]. Another study suggests that the C/S ratio is not the only governing parameter in determining E although it exhibits a slight overall decrease as C/S ratio increases [17]. A recent study, however, demonstrated that the modulus value of the C-S-H increases with the increase in the mean silicate chain length [18]. The bulk modulus of tobermorite was computed in two separate studies to be about 70 GPa [17,19].

### 2.2. Engineering C-S-H-based Nanocomposites

#### 2.2.1. Background

Environmental, socio-economic and modern engineering advances are contributing factors for sustainable development in the construction industry. Innovation has included global efforts to enhance the durability and performance of concrete structures. Strategies for achieving this goal include the fabrication of organic/inorganic nanocomposites where the continuous inorganic phase is calcium silicate hydrate-the principal binding component of cement-based products. Key goals include obtaining enhanced engineering properties (e.g., modulus of elasticity and strength) and improvement of durability. Organic moieties have been shown to be useful instruments for the nanostructural modification of C-S-H [20,21,22]. There appears to be a number of different interaction mechanisms, which are summarized below.

#### 2.2.2. Surface adsorption and grafting of polymers at defect sites

**Figure 3:**
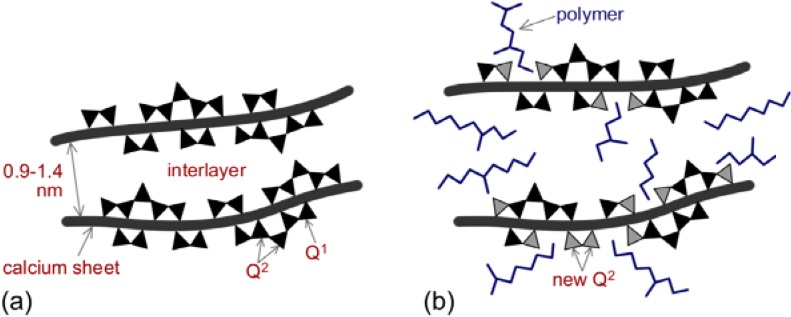
Schematic of polymer-modified C-S-H nanostructure. a: the nanostructure of pristine C-S-H. b: The nanostructure of modified C-S-H after the interaction with polymer molecules.

One possible mechanism is grafting at sites of missing silica tetrahedra on the silicate chain comprising C-S-H [22,23,24]. Analysis of the ^29^Si MAS NMR spectra indicate an increase in the Q^2^/Q^1^ ratio following the interaction of several organic molecules (e.g., hexadecylmethylammonium (HDTMA); poly(ethylene glycol) (PEG); poly(vinyl alcohol) (PVA); poly(acrylic acid) (PAA) and methylene blue (MB) [25]. This increase suggests that the chemical shift of silicon in the vicinity of the polymer can mimic that obtained with a Si-O-Si bond resulting in an apparent increase in the number of Q^2^ sites. This is illustrated by the schematic (Figure 3), which depicts the polymer modified C-S-H nanostructure.

The effectiveness of the grafting process (involving ionic or van der Waals forces) is dependent on the C/S ratio of the C-S-H as the number of defect sites generally increases with C/S ratio >1.0. It is possible that differences in reported results may occur due to differences in the preparation procedures of the C-S-H nanocomposites. The NMR evidence for a C-S-H-polymer interaction was supported by results from other analytical techniques.

#### 2.2.3. Intercalation of the interlayer spaces between the C-S-H sheets

It may also be possible for organic molecules to intercalate the interlayer spaces of C-S-H [26,27]. Evidence for the intercalation process is primarily based on the expansion of the basal-spacing (002) of synthetic C-S-H as indicated in the X-ray diffraction spectrum. The degree of expansion varied depending on the C/S ratio, synthesis methods (in-situ or exchange), type of molecules (non- ionic, anionic or cationic), concentration and pH [27]. Small shifts in the basal spacing of C-S-H could be interpreted as evidence of partial intercalation at layer ends [22,24].

#### 2.2.4. True hybrids and covalent bonding of polymers with C-S-H

True hybrids consisting of alkyl chains covalently bonded to the silicate sheets comprising C-S-H can also be formed [24]. The alkyl chains form a bi-layer in the interlayer space. The basal distance increases with the length of the alkyl chains. The process involves a co-operative assembly process of the inorganic and organic components during precipitation of the nanocomposites.

**Figure 4:**
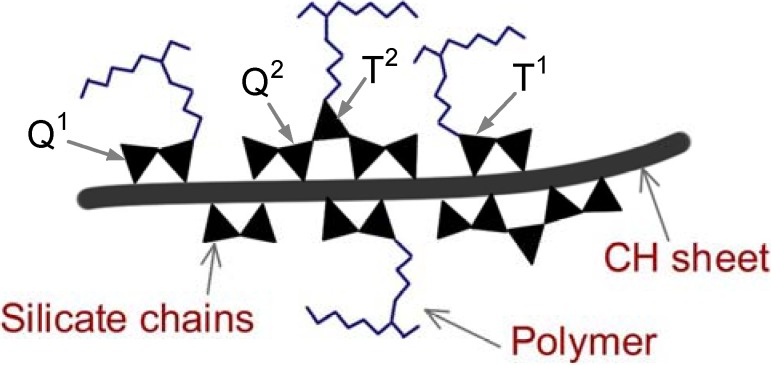
Schematic of polymer groups grafted at T silicon sites [28].

Intermediate substitutions of tetraethoxysilane (TEOS) by organotrialkoxysilane (OTAOS) resulted in the formation of C-S-H nanocomposites with the organic groups covalently bonded to the inorganic C-S-H framework, (Figure 4) [28]. Nanocomposites with polymer groups grafted at T silicon sites (*i.e.*, defect locations) can be formed by reaction of silylated polymers with OPC pastes (e.g., co-polymers of poly(dimethylacrylamide) (PDMA) and poly (butadiene-g-oxyethylene) (PBOE) silylated with T silanes) [28].

#### 2.2.5. Engineering properties and durability of C-S-H nanocomposites

Little information exists on the engineering performance of organic/inorganic C-S-H nanocomposites. Data indicating an apparent increase in the modulus of elasticity (40–100%) at low polymer content with respect to cement have been reported [28]. Quantitative information on the resistance to the transport of aggressive ions related to durability concerns needs to be obtained. Work is underway to evaluate the physical and mechanical stability of C-S-H nanocomposites and measure the time-dependent flow of helium into the interlayer spaces of C-S-H and polymer modified C-S-H to determine the relative ‘sealing’ capability of the polymer phase. The objective is to improve the resistance of C-S-H-based binders to the ingress of aggressive ions (e. g. sulfate) and minimize their interaction with the C-S-H phase.

## 3. Controlled Release of Admixtures

### 3.1. Background

Currently, there is an extensive use of chemical admixtures mainly to control/modify the fresh and hardened properties of concrete. The most common admixtures for cement and concrete include accelerators, set retarders, air entraining agents, and superplasticizers. Their successful use requires a basic knowledge of concrete technology, standard construction procedures, and familiarity with cement-admixture interactions. A particular challenge of interest to the authors is to optimize the use of dispersing agents such as superplasticizers in high performance concretes containing high volumes of supplementary cementing materials (SCMs). Dispersing agents such as superplasticizers are commonly used in these concretes. There are, however, practical problems such as loss of workability with time that are controlled by interactions with cement components. Controlling the timing of the availability of an admixture in cement systems is essential for its optimal performance.

Control release technology provides a route to prolonged delivery of chemicals while maintaining their concentration over a specific time period. In the field of medicine, a chemical is generally administered in a high dose at a given time, with dose repeated several hours or days later. This method is not economical and sometimes results in damaging side effects. As a consequence, increasing attention has been focused on methods of delivering drugs continually for prolonged time periods and in a controlled fashion. The primary method of accomplishing this controlled release has been through incorporating the chemicals within polymers [29], biodegradable nanoparticles [30], hydro gels [31], and other materials. This technology now spans many fields and includes pharmaceutical [32], and agricultural applications [33], cosmetics [34] household products [35], and more recently construction [36]. Here, a nanotechnology-based approach for controlled release of admixtures in cement systems using layered double hydroxides is presented.

### 3.2. Layered Double Hydroxides (LDHs)

**Figure 5:**
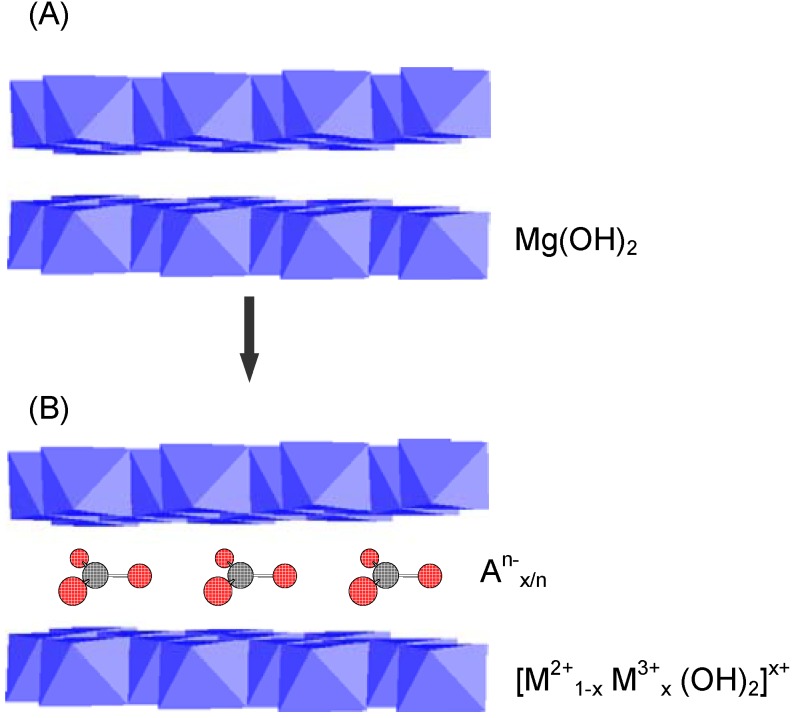
Schematic representation comparing the crystal structure of brucite (A) and LDH (B).

LDHs are a family of anionic clay materials, represented by the naturally occurring mineral hydrotalcite [Mg_6_Al_2_ (OH)_16_CO_3_·4H_2_O]. Most LDH minerals can be represented by the general formula: [M^2+^_1-x_ M^3+^_x_ (OH)_2_]^x+^[A^n-^_x/n_. mH_2_O]^x-^ where M^2+^ and M^3+^ are di- and trivalent metal cations (e.g., M^2+^ = Ni^2+^, Zn^2+^, Mn^2+^, Ca^2+^, *etc*.; M^3+^= Al^3+^, Ga^3+^, Fe^3+^, Cr^3+^, *etc.*), A^n-^ are interlayer anions of charge n, x is the molar ratio of M^2+^/( M^2+^+ M^3+^), and m is the number of moles of co-intercalated water molecules per formula weight of the compound. Structurally, cationic brucite-like layers are bound together by the interlayer counter anions as well as water molecules (Figure 5).

**Figure 6:**
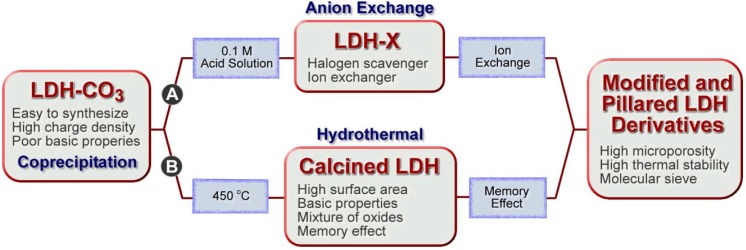
Summary of different paths to modified and pillared LDH derivatives.

In the past couple of decades, there has been a growing interest in the synthesis and use of LDHs and LDH-like materials. Because of their structural features, LDHs are easily synthesized by simple wet chemistry techniques and LDH-based materials can be produced via direct synthesis or most often indirect methods. Different paths to modified and pillared LDH derivatives are summarized in Figure 6.

Owing to the structural characteristics as well as the availability of simple synthetic methods, it is generally possible to tailor the physical and chemical properties of LDHs and LDH-based materials, which greatly helps produce functional materials with specific properties for targeted applications. They have been widely used as catalyst supports [37], flame-retardants [38], ions exchangers and absorbents [39], *etc*. New areas of applications have been discovered in the bio-medical field such as controlled drug delivery [40] and gene therapy [41]. In concrete science, LDHs and LDH-like materials represent one of the phases that form during cement hydration. Their relevance is highlighted in the next section.

### 3.3. Controlled Release Technology in Cement and Concrete

The LDH nanotechnology described in Section 3.2 applies to the applications detailed in the following Sections *i.e.*, Section 3.3.1 and Section 3.3.2.

#### 3.3.1. Interaction of superplasticizers with cement systems

The extensive use of cement and concrete and the frequent occurrence of kuzelite (a sulfate-bearing AFm phase) in hardened concrete [42,43] and in heavy metal-bearing phases [44,45], makes LDH and LDH-like materials good models to elucidate the interaction of organic admixtures with cement phases. The use of organic admixtures in cement-based materials influences the hydration process, the nature of the hydrated products, and consequently the mechanical properties of the hardened concrete [46,47,48]. It is generally acknowledged that the slump loss of fresh concrete at the construction site is one of the principal reasons associated with problems related to the strength and durability of concrete. Prolonged mixing in trucks accelerates stiffening of concrete and the rate and increase of slump loss. It is well documented that the addition of superplasticizers improves the workability and reduces the slump loss. However, the interaction of the admixtures with the aluminate phases, mainly 3CaO·Al_2_O_3_ (which is highly reactive and plays a key role in the early hydration reactions affecting the fluidity of the fresh paste immediately after mixing) may create problems. Adsorption, co-precipitation, and also intercalation within the structure of the hydrates formed at the early stage could be possible sources of undesired interactions, leading possibly to a loss of workability [49,50].

#### 3.3.2. Controlled release of admixtures

There have been a number of applications in cement and concrete where different means of controlling the effect of admixtures via a controlled release technique were used. A number of patents and research articles describe “encapsulation” procedures for delivery of liquids and solids. A corrosion inhibitor, such as calcium nitrite, was dispersed by encapsulation in coated hollow polypropylene fibers [51]. This anti-corrosion system was activated automatically when conditions would allow corrosion to initiate in a steel reinforced concrete. Porous aggregates were also used to encapsulate antifreezing agents [52]. Porous solid materials (e.g., metal oxides) have also been used as absorbing matrices to encapsulate chemical additives (e.g., accelerators, retarders, and dispersants) and to release them at a slower rate when combined with oil well treating fluids [53].

Another method to control the release of chemicals in cement-based materials is by “intercalation-de-intercalation”. A cement additive for inhibiting concrete deterioration was developed with a mixture of an inorganic cationic exchanger: a calcium zeolite capable of absorbing alkali ions (sodium, potassium, *etc.*) and an inorganic anionic exchanger: hydrocalumite capable of exchanging anions (chlorides, nitrates, sulfates, *etc.*) [54]. The results of their tests showed the potential of increasing concrete durability by exchange of alkali and chloride ions to inhibit alkali-aggregate reaction and corrosion of rebar.

More recently, work examined means to control the timing of the release of chemical admixtures through their incorporation in nanoscale composite materials [55,56]. More specifically, the technique consisted of intercalating an admixture into a hydrocalumite-like material, a calcium-based LDH derivative, and adding this composite to a cement-based mix. De-intercalation of the admixture can be actively programmed through controlled chemistry involving, for example, type of layered inorganic material, charge density, concentration, and/or pH. A sulphonated naphthalene formaldehyde-based superplasticizer, called Disal™ was used to produce the controlled release formulation (CaDisal) [56]. The effectiveness of Disal™ alone in controlling the slump-loss *versus* time characteristic was compared to that of the controlled release formulation CaDisal.

**Figure 7:**
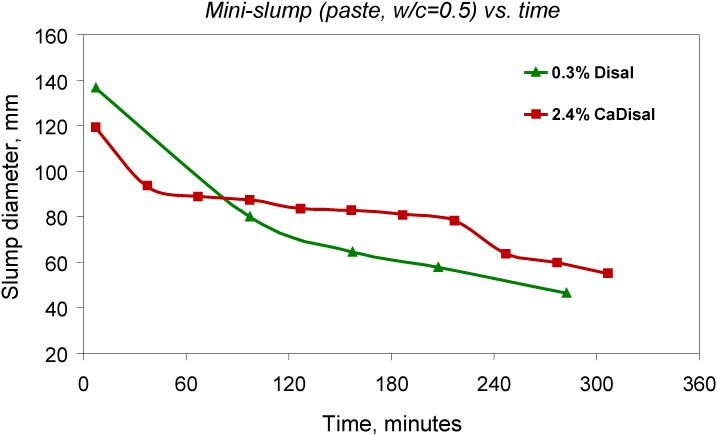
Slump loss with time for selected cement pastes.

Figure 7 is a plot of the slump loss of cement pastes produced with Disal™ alone (at 0.3% by mass of cement) and with the controlled release formulation CaDisal (2.4% CaDisal by mass of cement contains an equivalent amount of 0.3% Disal™) as a function of mixing time at room temperature using mini-slump measurements [57]. Mini slump is a measure of workability (flow and consistency) used for cement pastes. The slump typically decreases with time as hydration proceeds. The slump loss of cement paste produced with pure Disal™ and with the controlled release formulation CaDisal with respect to the elapsed time indicates that both samples have the same steep slump loss occurring in only 30 min for CaDisal as compared to 100 min for pure Disal™. It is important to notice that when CaDisal is used the magnitude of slump loss is considerably slower (due to controlled release) than when pure Disal™ is added to the mix. Overall, the plot indicates that the controlled release formulation provided a longer time for the superplasticizer to keep cement workability at a reasonable level after mixing. After 30 min of mixing, the workability of the mix is almost steady (plateau) up to 210 min. The controlled release formulation developed in the present work can therefore alleviate the difficulties associated with slump loss.

## 4. Carbon Nanotube/Cement Composite Materials

### 4.1. Mechanical Properties of Concrete

Concrete is a brittle material with a cement paste binder having a pore structure that contains micro (<2 nm in diameter) and fine mesoporosity (2–50 nm). Depending on its constituents, it can be very strong in compression (>200 MPa ultimate strength), but is generally weak in tension and flexure. It also has relatively low fracture toughness. These differences in strength are due as much or more to the internal pore structure of the concrete than the material itself. Typical concretes, made with mixtures of coarse rock aggregate, sand, OPC and/or other cementitious materials and water, have random packing of those materials that may produce a heterogeneous microstructure. In addition, the hydration reaction between the cementitious materials and the water does not produce an even growth of hydration products, which instead form a complex structure that becomes denser over time. This structure is generally less dense where it contacts aggregates, reducing the bond between the cement matrix and providing a route for cracks to propagate.

In traditional concrete, weakness in tension and flexure is overcome by designing to minimize the effects of those forms of loading and by the addition of reinforcing steel to provide adequate strength when the loads do take place. The reinforcing steel rods serve to accommodate the tensile stresses in flexural members. Pre-stressing steel placed under tension keeps the surrounding concrete under compression to prevent cracking due to loading.

### 4.2. Fibers and Nanotube Reinforcements

Although these traditional approaches described above have been very successful, they are limited both in terms of preventing local cracking in the concrete matrix and in allowing the design of structures capable of dealing with high flexural loadings. Fiber reinforcements have been used in concrete to try to overcome these limitations [58]. With typical lengths in the range of 1–10 centimeters and diameters from 0.1 to 1 mm, commercially available fibers increase flexural strength. They also interrupt crack propagation much more quickly than do standard reinforcing methods, which should improve the fracture toughness of the material. In some applications these fibers can completely replace standard reinforcements, while in other they are used together [59].

The use of carbon nanotubes (CNT) as a reinforcing material is intended to move the reinforcing behavior from the macroscopic to the nanoscopic level. In addition to the well known advantages of these materials as reinforcements, which include extremely high strengths [60] and Young’s moduli [61], elastic behavior [62] and advantageous electronic [63] and thermal properties [64], in the mesoporous environment of concrete, nanoscale reinforcements hold the potential to act as fillers, producing denser materials; to inhibit crack growth at very early times, preventing propagation; and to enhance quality of the paste-aggregate interface. As a result, much stronger and tougher concretes may be possible when made as a CNT composite.

### 4.3. Development and Properties of CNT/OPC Composites.

#### 4.3.1. Dispersion of CNT

While there has been considerable effort directed toward the development of a variety of CNT composites, OPC composites offer unique challenges with respect both to dispersion and formation of the composite itself. In polymeric composite materials the CNT may be fiber dispersed in a solvent suitable for the production of the composite, with or without functionalization. In metallic or ceramic composites, mechanical methods such as co-grinding may be employed. Neither approach is entirely applicable to OPC composites. Typical solvents used for polymer composites may have adverse effects on OPC hydration properties, while the relative softness of some OPC constituents such as gypsum means that CNT would be unlikely to become well dispersed using a grinding process. Instead, researchers have focused on methods of dispersal that are compatible with OPC chemistry. The primary approach has been to use commonly used admixtures such as water reducing admixtures and superplasticizers as dispersing agents. Research to date suggests that dispersion in polycarboxylate or in solutions of polyacrylic acid can be successfully achieved for multiwalled CNT (MWCNT) [65,66,67,68] while napthalene sulphonates have been successful for single walled CNT (SWCNT) [69]. In some cases the MWCNT have been functionalized by the attachment of carboxylic acid groups to aid dispersion [65,66,68], but this approach has not been found to be necessary by all researchers. The precise mechanism of dispersion has yet to be established for CNTs placed in these admixtures.

Another dispersion method used in recent work on SWCNT [69,70] was to sonicate the OPC and CNT together in isopropanol and then allow the liquid to dry. A disadvantage of this method is that the OPC grain surface is damaged by the process of sonication, slowing down the initial hydration process in samples without SWCNT. It is also affected by the method of sample purification used by the SWCNT manufacturer, with the success of the dispersion dependent on the sample source [69].

#### 4.3.2. Composite properties

As with early work on other CNT composite materials, the results of mechanical testing of OPC/CNT composites have been highly variable, with some tests producing significant improvements in compressive strength, Young’s modulus and hardness, while others giving inconsequential changes in compressive strength or significant decreases in Young’s modulus. The best observed performances include a 50% increase in compressive strength in a MWCNT sample [68], over 600% improvement in Vickers’s hardness at early ages of hydration for a SWCNT sample [69] and a 227% increase in Young’s modulus for a MWCNT composite sample [66]. Results to date have not convincingly shown improved flexural strength, with those samples showing improvements having too short an aspect ratio to give purely flexural behavior.

**Figure 8:**
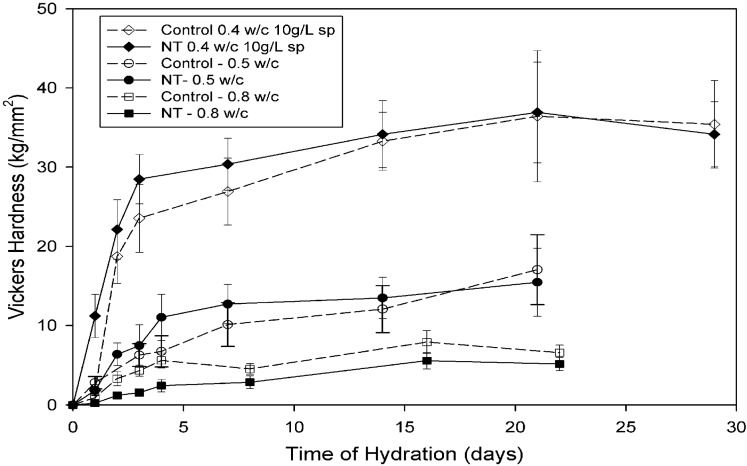
Hardness measurements on cement compacts made at different water/cement ratios with (NT) and without (control) 2% SWNT additions [69]. “sp” indicates that the sample was prepared with a napthalene sulphonate superplasticizer.

A survey of these results strongly suggests that improvements in composite performance require very specific combinations of admixtures, water to cement ratio and nanotube content. A study of fifteen different combinations of MWCNT, admixtures and w/c ratios found one that produced significant improvements in strength as compared to a control sample [68]. Similarly, work on nanoindentation showed Young’s modulus improvements for single samples of MWCNTs and SWCNTs [66], with remaining samples showing significant decreases in performance. In addition, work on SWCNTs [69] has suggested that the observed improvements in behaviour were dependent both on sample composition and hydration time. Samples produced at 0.8 w/c ratio, showed reductions in strength at all time periods. In contrast, low w/c samples with a 2% SWCNT content showed improvements of up to 600% in hardness at early ages, but essentially no improvement after 14 days of hydration (Figure 8).

**Figure 9:**
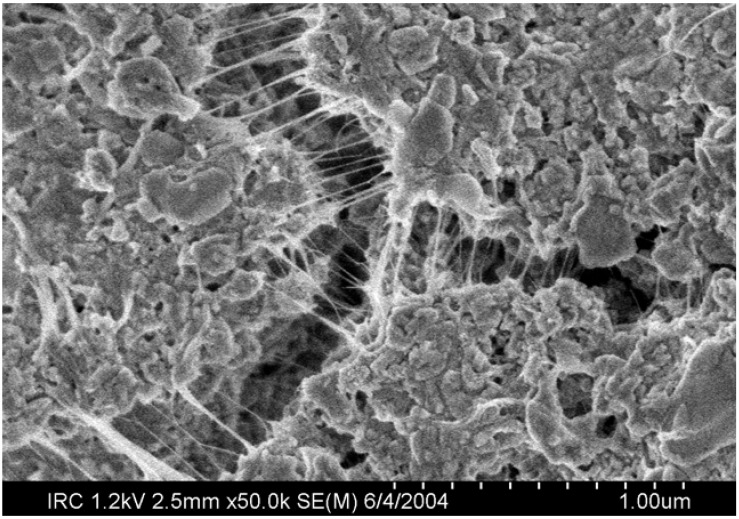
Example of crack bridging in a SWCNT/hydrated OPC composite bridging structures are SWCNT bundles.

**Figure 10:**
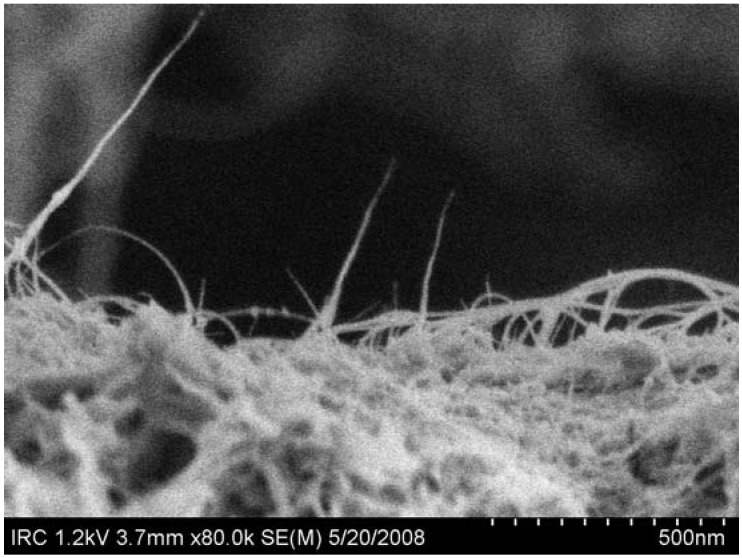
SWCNT bundles on a hydrated OPC surface after pullout.

While measurements on bulk samples have been inconsistent, investigations of microstructure have indicated that if the nanotubes are well dispersed there may be potential for improving the mechanical properties of cement-based materials in a more consistent way. Fourier Transform-Infrared Spectroscopy [65] has shown that chemical bonding can be obtained between the carboxylated MWNT and the cement matrix. Evidence for crack bridging in functionalized MWCNT composites has been observed [65,68]. Significantly, crack bridging is readily observable (Figure 9) in unfunctionalized SWCNT composites. Other forms of classical reinforcing behaviour such as fiber-pullout (Figure 10) and crack deflection have also been observed.

In addition to the mechanical studies, work has been undertaken on the electrical properties of MWCNT/cement composites. Although the researchers found significant improvements in DC electrical conductivity as compared to control samples, the measured values, on the order of 130–170 ohm-cm [71,72], fall far short of the level of conductivity that should be expected based on the level of loading of MWCNT in the samples. However, AC impedance measurements [72] showed that in comparison to measurements on samples produced with carbon fibres, the MWCNT produce low imaginary impedance values, an indication that the MWCNT were contributing directly to the impedance spectra as conductors. The result that MWCNT acted as conductors without producing the expected reduction in conductivity suggests that there was little direct connectivity between the MWCNT in the hydrated OPC samples.

#### 4.3.3. Interactions between OPC and CNT

**Figure 11:**
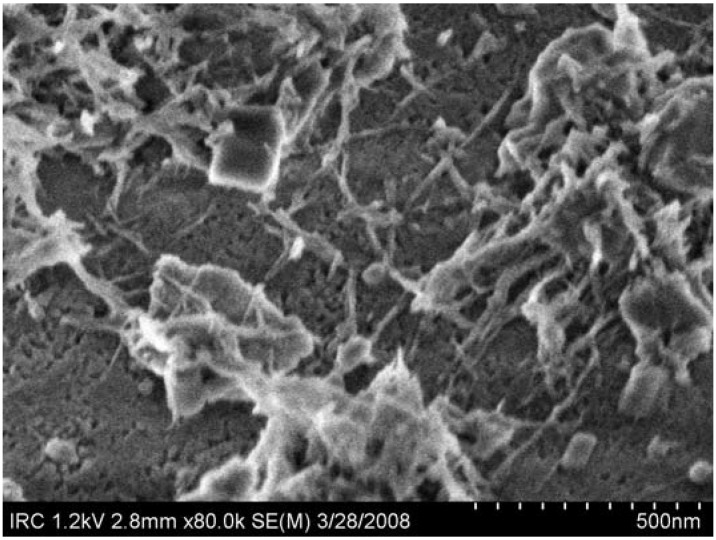
Growth of C-S-H around SWCNT bundles (rods in image) at 135 minutes of hydration of an OPC composite sample.

Both the electrical behavior of the composite material and the observed microstructural properties can be understood through an examination of the effects of the CNT on the hydration processes of the OPC. Recent work [70] has shown that SWCNT act as nucleating agents for C-S-H, which preferentially form on the surface of nanotube bundles as opposed to the surface of the adjacent unhydrated cement grains. The nucleation appeared to occur along the entire length of the SWCNT, rather than at specific locations that might be associated with functional groups (Figure 11). The result was a dense C-S-H formation that appears to be tightly bonded to the SWCNT, producing reinforcing behavior. Formation of C-S-H around functionalized MWCNT has also been observed [71], but it is likely that the same processes observed in SWCNT also occur in MWCNT systems. This suggests that the lack of electrical connectivity observed in those systems [72] is directly due to C-S-H preventing contact between the nanotubes.

## 5. Nanoparticles Technology

### 5.1. Background

Nanoparticles technology, which covers synthesis, process, characterization and application of nanoparticles, currently attracts considerable attention because of a wide variety of potential and practical application, including medicine, electronics and advanced ceramics. In general, a nanoparticle has a particle size in the range of 1 to 100 nm. The distinctive properties of nanoparticles are significantly different from those of the bulk materials. The application of nanotechnology became a key topic in the field of cement and concrete in the last decade, and consequently the nanoparticle technology started to play an important role as well.

### 5.2. Nanoparticles in Cement and Concrete

The most widely conducted studies on the use of nanoparticles in cement and concrete have been on nano-oxides, especially SiO_2_ and Fe_2_O_3_ [73,74,75,76,77,78,79,80]. The addition of these nanoparticles to cement paste containing high volumes of fly ash and to sludge ash concrete mortars resulted in an increase in compressive strength. Nano-Fe_2_O_3_ and nano-SiO_2_ were also used to increase the abrasion resistance of concrete for pavement [78]. Nano-Ca(OH)_2_ particles have been prepared and their thermal properties were characterized to study the anomalous behaviors of Ca(OH)_2_ in cement paste [81]. Also, other nano to sub-micro inorganic particles, such as zeolite, have been added to cement systems with the goal to improve the overall microstructure [82]. Another nanosize oxide of interest to construction is TiO_2_. It has recently been reported that the TiO_2_ nanoparticles accelerated the rate of hydration and increased the degree of hydration [83]. Its photocatalytic characteristics have been mainly used to remove organic pollutants from surfaces directly exposed to ultraviolet radiations such as road pavements and cement-based façade finishing products [84]. Synthetic C-S-H has also been used as seeding agent during the hydration of cement phases [85]. The possibility of controlling the nature of hydration products through nucleation seeding of different types of pre-formed C-S-H was demonstrated.

### 5.3. Research on CaCO_3_ Nanoparticles—A Case Study

The use of high volumes of SCMs such as fly ash and blast furnace slag as replacements for OPC in concrete can have environmental as well as economic benefits, through the reduction of green house gases from the production of OPC, diversion of the SCMs from landfills and reduced use of the natural resources used to manufacture OPC. The appropriate use of SCMs can also improve concrete properties and increase the service life of concrete structures. One drawback of using high volumes of SCMs is a resulting delay in initial setting time, which reduces early strength of concrete. However, recent work has shown that nano-CaCO_3_ can have a significant, beneficial impact on the hydration of OPC/SCM blends [86,87] as compared to micro-CaCO_3_, potentially solving this roadblock to reducing greenhouse gas production in the construction industry.

The use of CaCO_3_ was first considered as a filler in cement to replace OPC. However, the results from a number of studies have shown positive effects of CaCO_3_ additions in terms of strength and acceleration of hydration rate. A study on the accelerating effect of finely ground CaCO_3_ addition on the hydration of C_3_S showed that the higher the CaCO_3_ addition, the greater was the accelerating effect [88]. The accelerating effect of the finely ground CaCO_3_ addition on the hydration of cement paste was also observed [89].

**Figure 12:**
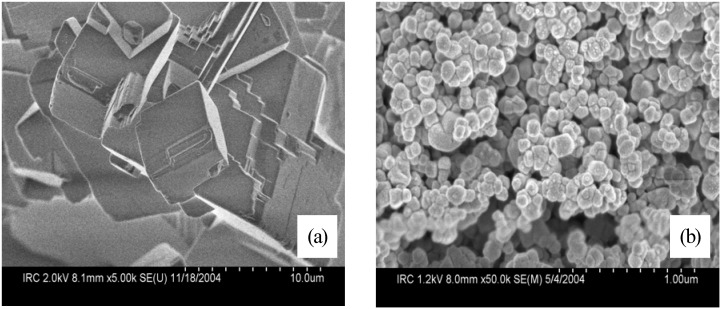
Scanning electron microscope image of (a) micro-CaCO_3_ and (b) nano-CaCO_3_.

**Figure 13:**
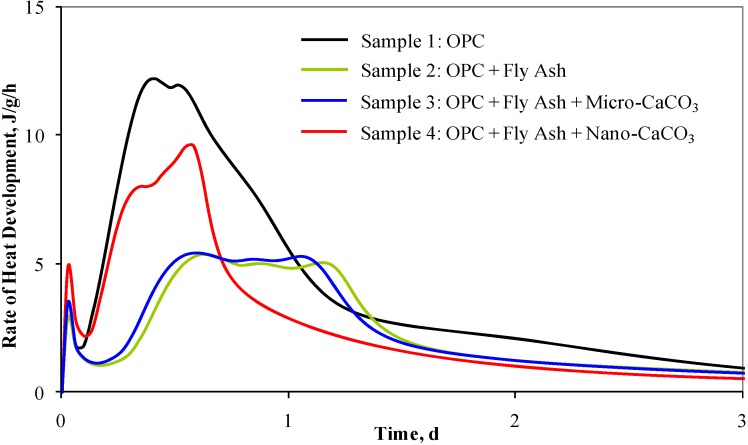
Rate of heat development measured by the conduction calorimeter.

Scanning electron microscope (SEM) images of both types of CaCO_3_ particles are shown in Figure 12. The average particle size of the micro-CaCO_3_ was approximately 5 to 20 µm whereas that of nano-CaCO_3_ was about 50 to 120 nm. The surface area values of micro-and nano-CaCO_3_ were 0.35 m²/g and 20 m²/g, respectively. Different cement pastes with variable additions of fly ash and nano-CaCO_3_ were prepared and analyzed. Figure 13 illustrates the rate of heat development measured by the conduction calorimeter for four different samples. The rate of heat development of the 50% OPC and 50% fly ash blend is significantly lower than that of 100% OPC (sample 1). When micro-CaCO_3_ was added to the OPC/fly ash blend (sample 3), a slight acceleration above that of sample 2 was observed. When nano-CaCO_3_ particles were incorporated (sample 4), the rate of heat development was significantly accelerated.

**Figure 14:**
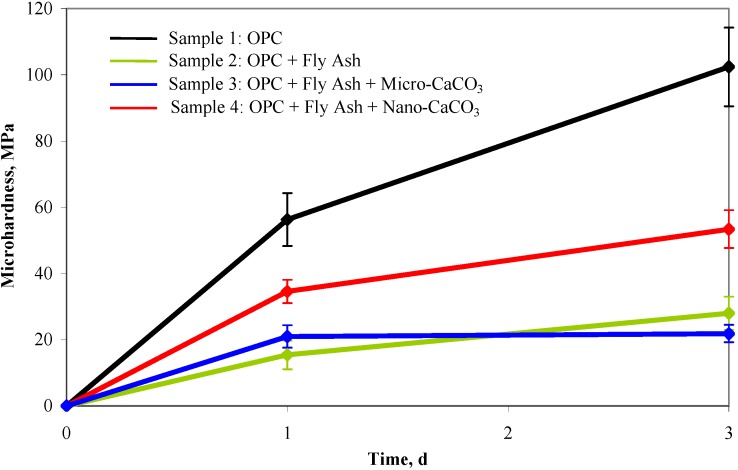
Microhardness measurement for the hydration of 1-day and 3-days.

Microhardness tests were performed by indentation under static loading (Figure 14) on the same blends of materials after 1 and 3 days of hydration. The results showed a significant improvement in the strength when nano-CaCO_3_ is added to OPC/fly ash blend. The microhardness value at 1–day hydration for 100% OPC reached 56 MPa but dropped to 15 MPa for 50% OPC and 50% fly ash blend, with a similar decrease for the blend with micro-CaCO_3_. However, when 20% nano-CaCO_3_ was added to the OPC/fly ash blend, the microhardness value increased to 35 MPa. At 3–day hydration, the microhardness value for the OPC/fly ash blend was again increased with the 20% addition of nano-CaCO_3_ (sample 4) as compared to OPC/fly ash blend alone or with micro-CaCO_3_. These observations clearly indicate the efficacy of using nano-CaCO_3_ (as opposed to micro-CaCO_3_) in improving the initial strength development of OPC containing high volumes of SCMs.

## 6. Economic and Related Factors Affecting the Application of Nanotechnology to Concrete

### 6.1. Acceptance of Nanotechnology in the Construction Industry

Concrete is the single most commonly used construction material in the world. More than 2.6 billion tons of OPC was produced worldwide in 2007 [90], corresponding to more than 17 billion tons of concrete. This production of OPC was used in a wide range of products, ranging from basement foundations and house walls through road pavement and pre-cast lamp poles to bridges, dams and high rise towers. Concrete products are generally intended to have long life-spans and be resistant to local environmental conditions. However, in most cases they will eventually be demolished and possibly recycled when they reach the end of their service lives.

In addition, the nature of the construction industry is such that it is easier to implement process innovations, rather than disruptive product innovations. Construction integrates products from a wide range of suppliers and skills from a wide range of contractors, subcontractors and trades into a single finished structure. A change in the way a structure is built can be determined by the construction company itself, but a significantly new product needs to be created by a supplier, understood and approved by architects, engineers and the client and implemented by on-site workers who may need to be specifically trained in its use.

All of these factors must be taken into consideration in developing nanotechnology for use in concrete. First, concrete and related products are bulk commodities. Even high value concrete structures require low materials costs and the ability to handle large quantities of material in a safe and environmentally acceptable manner. Second, innovations need to be thoroughly developed and field tested in order to build the knowledge and confidence in the construction community. Finally, concrete structures can be difficult to demolish, often requiring explosive or other high-energy approaches as an initial step to break up the major components of the structure. Nanotechnology used in concrete must therefore be compatible to these traditional practices.

Given these constraints, the initial nanotechnology applications in construction are those that provide a clear benefit in terms of added functionality with relatively small amounts of nanomaterials that can be delivered in using standard construction practices and will not affect other aspects of the performance of the material. Novel products that improve the delivery of existing materials, such as the control released admixture work described here, are likely to be next to market. Other innovations, such as nanocalcium carbonate accelerators, will become more common as the price of the nanomaterial falls to the point where it can be used in bulk. Carbon nanotube/cement composites, in contrast, will likely take the longest time to implement as they will require further fundamental research, reductions in CNT prices, the development of specialized delivery techniques and equipment, greater understanding of the environmental impact of CNTs and specialized demolition methods before wide spread adoption can occur.

### 6.2. Potential Economic Impact of Nanotechnology on the Construction Industry

The emergence of nanotechnology applications in the area of concrete construction is relatively recent and many developments are still in the commercialization process. However, the research described here has economic implications ranging from the indirect impact of improved understanding of the performance of cement and concrete to new products that are in the process of becoming market ready. In terms of basic science, improvements in the understanding of the characteristics of the hydration products of Portland cement (particularly C-S-H) at the nanoscale should facilitate the more efficient manipulation of the nature of cement-based materials. For example, current research has demonstrated that the incorporation of nano-sized C-S-H particles as seeds into cement systems can be used to tailor the overall composition of the C-S-H and monitored using newly developed quantitative ^29^Si MAS NMR techniques [85]. This makes possible the development of a new generation of concretes that can address specific durability and environmental concerns. Commercial products comprised of C-S-H seeding material are currently available. The economic implications are significant in terms of improvements in life-cycle performance and maintenance costs.

The economic impact of carbon nanotube/cement composite materials is restricted by the high expense of the carbon nanotubes. Even at very low rates of addition, current prices of carbon nanotubes are high enough that the production of significant composite structures is cost prohibitive. Even with large reductions in nanotube prices, the composite material is most likely to be used in niche applications, where the requirements for ultra-high toughness and strength compensate for the nanotube cost and the potential difficulties associated with end of service life demolition. Bridges, nuclear reactor containment vessels, dams in high risk locations and military facilities are among the potential applications.

The ability of nanoparticle additions to accelerate the rate of hydration of OPC blends opens the possibility of significantly lowering the content of cement in concrete. High supplementary cementing material (SCM) contents in blended cements can drastically slow hydration, but the effect can be compensated by the presence of a nanoparticle accelerator. A key factor to economic viability is the need for the cost of the nanoparticles to be lower than the cost of the cement that is replaced. This condition in turn implies that much lower nanoparticle contents should be used in the blend than the percentage of cement that was replaced. Providing this condition is met, significant economic benefits are possible through reduction of the amount of cement used in a structure. In addition, increasing the possible percentage of SCMs in OPC blends should produce strong environmental benefits due to the reuse of waste materials and reductions in the greenhouse gases associated with OPC production.

Finally, commercial applications for the controlled release (CR) technology are numerous. In concrete construction, they involve in-situ, real-time delivery of accelerators, retarders, superplasticizers and other concrete admixtures used in oil well cements, cast in place, and precast concretes. The interaction of the commercial chemicals with cement minerals and hydration products can therefore be controlled during the hydration process, resulting in improved performance of both admixtures and the resulting concrete products.

The examples discussed here show that the range of potential economic outcomes from the application of nanotechnology to cement and concrete reflects the diversity of those applications. The overall potential is, however, very high. Nanotechnology offers a route to precisely engineer concrete for specific applications, reducing costs and increasing performance. Entirely new applications for concrete are also possible. Developing cement and concrete related nanotechnology will therefore have a sustained and important impact on the future of the construction industry.
